# Affective Norms for Italian Words in Older Adults: Age Differences in Ratings of Valence, Arousal and Dominance

**DOI:** 10.1371/journal.pone.0169472

**Published:** 2017-01-03

**Authors:** Beth Fairfield, Ettore Ambrosini, Nicola Mammarella, Maria Montefinese

**Affiliations:** 1 Department of Psychological, Health and Territorial Sciences, University “G. d’Annunzio”, Chieti, Italy; 2 Department of Neuroscience, University of Padua, Padua, Italy; Boston Children's Hospital / Harvard Medical School, UNITED STATES

## Abstract

In line with the dimensional theory of emotional space, we developed affective norms for words rated in terms of valence, arousal and dominance in a group of older adults to complete the adaptation of the Affective Norms for English Words (ANEW) for Italian and to aid research on aging. Here, as in the original Italian ANEW database, participants evaluated valence, arousal, and dominance by means of the Self-Assessment Manikin (SAM) in a paper-and-pencil procedure. We observed high split-half reliabilities within the older sample and high correlations with the affective ratings of previous research, especially for valence, suggesting that there is large agreement among older adults within and across-languages. More importantly, we found high correlations between younger and older adults, showing that our data are generalizable across different ages. However, despite this across-ages accord, we obtained age-related differences on three affective dimensions for a great number of words. In particular, older adults rated as more arousing and more unpleasant a number of words that younger adults rated as moderately unpleasant and arousing in our previous affective norms. Moreover, older participants rated negative stimuli as more arousing and positive stimuli as less arousing than younger participants, thus leading to a less-curved distribution of ratings in the valence by arousal space. We also found more extreme ratings for older adults for the relationship between dominance and arousal: older adults gave lower dominance and higher arousal ratings for words rated by younger adults with middle dominance and arousal values. Together, these results suggest that our affective norms are reliable and can be confidently used to select words matched for the affective dimensions of valence, arousal and dominance across younger and older participants for future research in aging.

## Introduction

Emotionally laden words influence a number of cognitive processes, such as lexical decision [1–4], reading [5,6] and memory [7,8]. More importantly, the affective appraisal of words varies from one culture to another [9], as well as between languages [10]. As a consequence, researchers have spent much effort in developing affective norms for words in a great number of languages, such as Italian [11], English [12–14], German [15–18], French [19,20], Finnish [21], Portuguese [22], Dutch [23], Brazilian [24], Spanish [25,26] and Chinese [27], in order to specifically characterize the affective content of words for each language. In this vein, it is not surprising that in the last year alone a host of affective norms for verbal stimuli have become available [27–36].

As posited by the dimensional theory of emotion [37], the affective connotation of words can be described along a number of different dimensions. This view is based on the seminal study of Osgood [37], who applied factor analyses to a wide variety of verbal differential judgments. In general, this analysis showed that two main factors, valence and arousal, explained the major portion of variance in affective meaning, suggesting that individuals approach pleasant or positive stimuli and avoid unpleasant or negative ones with variable degrees of intensity. A third dimension, called dominance or control, although significant, explained a smaller amount of variance. The term *valence* indicates the way an individual judges a stimulus (unpleasant vs. pleasant), the term *arousal* indicates the degree of activation an individual feels towards a stimulus (calm vs. exciting), whereas the term *dominance/control* indicates the degree of control an individual feels over a given stimulus (out of control vs. in control).

In spite of the large effort made to collect language-specific affective norms for words, little effort has been given to developing age-specific affective norms [20,38–40]. The lack of affective norms in older participants has obliged researchers to study the impact of age on emotion, by adopting stimuli derived from norms on younger adults or collecting affective ratings on older adults a posteriori. Indeed, standardized ratings in an older population are fundamental for selecting stimuli in an initial phase of any experimental study (see [41]).

Moreover, recently the effects of aging and affective information processing has generated considerable interest in researchers especially since older adults seem to have preserved functioning in emotion processing compared to younger adults [41]. In fact, studies concerning memory [42,43], attention [44], and decision-making [45] have repeatedly shown how older adults regulate their cognitive resources towards positive emotional information more than negative information in order to maximize positive affect and minimize negative affect compared to younger adults [46]. Moreover, this effect has been observed for different affective stimuli, including words [47], pictures [42], and faces [44]. More importantly, this growing amount of literature also highlights how older adults experience affective content differently than younger adults. In particular, a study by Kensinger and colleagues [48] found an age-related interaction between perceived affective content in words and word recall from memory when participants’ own affective ratings were used whereas no significant interaction between age and valence in memory performance was found when younger adults’ ANEW ratings were used. The reversed pattern was observed in younger adults. These results suggest that age effects on the perception of affective content in stimuli are crucial and must be taken into account when studying the cognitive processing of these stimuli.

In line with this, Grühn and Smith [38] asked older and younger adults to rate 200 German adjectives in terms of valence and arousal and found that older adults rated positive words as more positive than younger adults. Differently, Keil and Freund [49] compared ratings for 90 German verbs between younger adults, middle-aged adults and older adults and found that the older adults gave more negative ratings for negative words than the younger adults, but they did not find age-related differences for positive words.

Aging also affects the emotional arousal or intensity of emotional processing, with a mixed pattern of results. For example, older adults typically have difficulties in regulating physiological arousal and inhibiting the processing of high-arousing material [50]. Older adults also tend to report experiencing less intense emotion in general than young adults [51]. By contrast, Smith and colleagues [52] suggested that emotional arousal increases with age while other studies found slight or no differences in arousal ratings between age groups [53–55].

More importantly, older adults have shown an additive effect of valence and arousal on stimulus processing in a different way from that of younger adults. Indeed, the relationship between valence and arousal is best described as a U-shaped curve in younger adults, where items with less extreme (i.e., moderate) valence ratings are perceived to be the least arousing, whereas items with more extreme (i.e., highest and lowest) valence ratings are perceived to be the most arousing [11,14,23]. On the contrary, different results have been proposed for older adults. Older adults, who perceived negative stimuli as more negative and arousing and positive ones as more positive and less arousing than younger adults, present a strong linear relationship between valence and arousal [38,46,49]. Older adults also gave higher arousal ratings for negative words than younger adults did [39]. For example, a study by Backs and colleagues [53] found age differences in affective dimensions using the Self-Assessment Manikin (SAM, [56]) -a nonverbal pictographic measure. Younger adults rated pleasant-arousing stimuli as more pleasant and arousing than older adults while older adults rated the pleasant-calm stimuli as more pleasant than younger adults, suggesting that compared to younger individuals, older adults experience greater emotional regulation (with a lower affect intensity) and a leveling of positive affect.

Lastly, neuroimaging studies have shown how normal aging leads to changes in the neural substrates involved in valence processing. In particular, a study by Kehoe and colleagues [57] found that older adults showed greater activation in the left amygdala, left middle temporal gyrus and right lingual gyrus in response to positive valence while, on the contrary, they showed reduced reactivity to emotional arousal, in occipital and temporal visual cortices, the left inferior parietal cortex and in bilateral supplementary motor areas.

Age effects on dominance ratings are even more limited than for the other two affective measures. To the best of our knowledge, only one normative study has investigated age effects on the dominance dimension [38]. The authors revealed that older adults rated positive adjectives as more arousing and less controllable than younger adults did.

Clearly, emotional stimuli need to be standardized taking into consideration age-related effects in order to untangle the complex interaction between cognition and emotion. However, as of yet, affective norms for words for the aging population are missing in Italian. Therefore, the main aim of our study was to create a standardized version of the affective word database of the 1121 Italian words in the adaptation of the Affective Norms for English Words (ANEW) for Italian [11] for older adults. The secondary aim was to investigate how the processing of affective information of words changes with age. To this end, we compared subjective responses to the affective content words in terms of valence, arousal and dominance of older adults with that of younger adults derived from our previous affective norm [11].

The importance of developing an Italian database for affective words in older adults is two-fold. First, it may foster cross-cultural studies using common methodologies. That is, it may stimulate specific databases for affective words in older adults in other languages as well. Second, it will allow researchers to select proper standardized stimuli in experimental studies on older adults and thus may promote research on this population.

## Materials and Methods

### Participants

A total of 240 right-handed older adults (120 females; mean age: 69.36 years, *SD* = 7.73 years; mean education: 9.26 years, *SD* = 5.26 years) participated in our study. Their Mini-Mental State Examination (MMSE, [58]) was higher than the cut-off score 24 (*M =* 27.06, *SD* = 2.79), showing no cognitive impairment. All participants were Italian native speakers, had normal or corrected-to-normal vision, were naïve as to the purpose of the study and gave written informed consent prior to their inclusion in the study. The study was performed in accordance with the ethical standards of the 2013 Declaration of Helsinki for human studies of the World Medical Association and was approved by the Departmental Ethics Committee of the University of Chieti.

### Materials and procedure

The stimuli set was composed of the affective words normed in the Italian adaptation of the ANEW on young participants [11]. In particular, the data set included the Italian translation of all the English ANEW words (1,034 words) [12] and those from the Italian semantic norms collected in our laboratory (87 words) [59,60] and tested in behavioral [61,62] and psychophysiological [63] studies.

As for the affective norms on young participants, participants rated their affective state on the following affective dimensions using the 9-point SAM scale [56]: 1) valence -the feeling of pleasantness elicited by a given stimulus- from *very unpleasant* (1) to *very pleasant* (9); 2) arousal –the feeling of excitation elicited towards a given stimulus- from *very calm* (1) to *very aroused* (9); 3) dominance –the feeling of control over a given stimulus- from *very submissive* (1) to *very dominant* (9).

Word stimuli were distributed over 10 lists containing 112–113 words each. In each list, words were randomized across participants in order to avoid primacy or recency effects. All lists were matched for valence (*M* = 5.21, *SD* = 2.06), arousal (*M* = 5.64, *SD* = .91) and dominance (*M* = 5.23, *SD* = .98) indexes derived from the Italian affective norms [11].

Participants were given an 8-page A5-format booklet with written instructions at the top on the first page. They were instructed to rate how they felt when reading the words along the three affective dimensions by means of SAM (for further details on the stimuli and procedure, see [11] and the *Instructions* sheet of S1 Database). Noteworthy, we used the exact same terms used in Bradley’s original study to describe the extreme values of the scales. A researcher controlled that the participants clearly understood the instructions and had no doubts about the rating procedure; additional verbal instructions and clarifications were given whenever necessary.

## Results and Discussion

### Database

The affective norms for Italian words in older adults’ corpus, available as S1 Database, contains 1,121 Italian words with normative data on affective ratings of valence, arousal and dominance provided by 240 older adults (see Participants section). Each word was rated by 24 older adults (twelve females and twelve males). The present set of words is the same as that in the Italian adaptation of the ANEW in young participants [11]. There were a few missing values for all three affective measures because some words meanings were unknown to a few older adults (.51% of the total responses).

### Descriptive statistics

**Fig 1:**
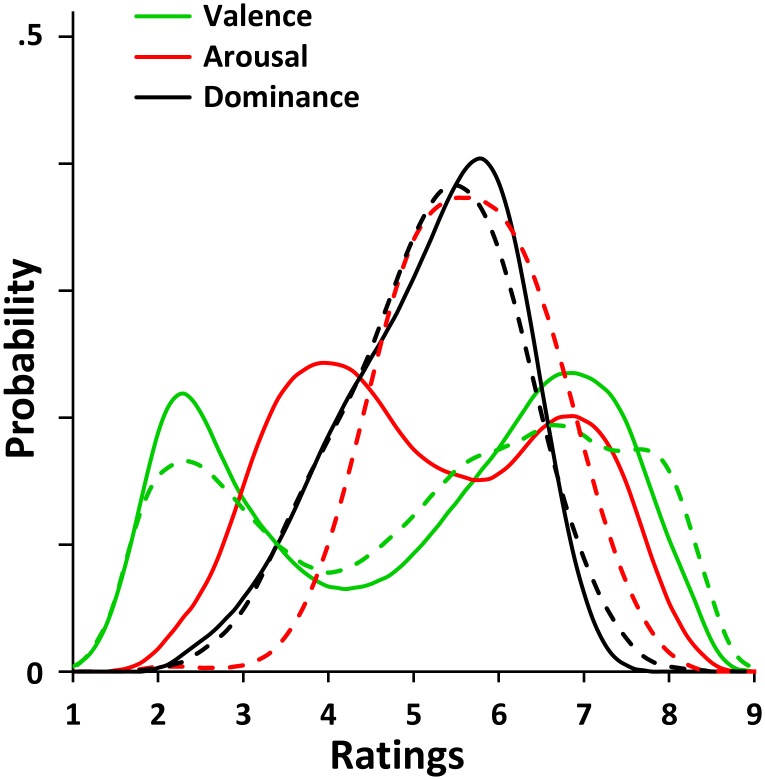
Distribution of affective ratings. The figure shows the kernel smooth density plot for mean affective ratings in older participants (solid lines). The distributions of affective ratings in younger participants from our previous affective norms [11] are also shown (dashed lines) for comparison.

Fig 1 shows the distributions of the mean ratings for the three affective dimensions for older participants (solid lines). The three distributions deviated significantly from a normal distribution (Kolmogorov-Smirnov test: *D*s > .080, *p*s < .01). Kurtosis was -1.40 for valence, -1.15 for arousal and -.32 for dominance dimension, indicating a relatively flat distribution compared to the normal model. Skewness was slight negative for valence (-.26) and dominance (-.56) and slight positive for arousal (.12).

These results suggest a slight positivity bias for the valence dimension; this was confirmed by the fact that 57% of the words was rated as being above the median value (i.e., 5), a percentage that was statistically different from chance level (*p* < .001, binomial test against 50%). This result suggests that older people present a bias towards positive words that is similar (χ^2^ (1) = .530, *p* = .467) to that shown by younger participants ([11], see also [14]), who rated 59% of the words with above-median values in our previous study. This would reflect “the preference of humankind for pro-social and benevolent communication” (p. 1194, [14]) and the prevalence of positive words in Italian as in other languages [64,65]. A stronger bias toward the high range of the scale was observed for the dominance dimension, as 61% of the words were rated above 5 (*p* < .001, binomial test), whereas no evident bias was found for the arousal dimension, as 49% of the words were rated above 5 (*p* > .338, binomial test).

Interestingly, unlike the distribution of dominance, which was clearly unimodal (Hartigan’s dip test of unimodality [66]: *p* = .492), valence and arousal were bimodal in our sample (dip test: both *p*s < .001). These results corroborated those obtained for words from younger [11,14] and older participants for dominance [38]. Moreover, they partially confirm our results on the younger population for valence and arousal. Indeed, some affective norms have found a bimodal distribution for valence in younger [11,14,23] and older samples ([38,39] but see also [20]). To the best of our knowledge, no previous affective norm has revealed a bimodal distribution for arousal dimension. Bipolarity for valence and arousal distributions have been postulated in past literature (see the circumplex model, [67]) and is still a key issue in current research.

### Reliability of measures

We evaluated the consistency of the data collected within each of the three affective ratings by means of split-half correlations corrected with the Spearman-Brown formula. All reliability coefficients were calculated on 5000 different randomizations of the participants in two subgroups of equal size. The median correlations between the two subgroups were very high for all dimensions, revealing that our ratings were very consistent. Specifically, the mean correlation for valence was .98, ranging from .97 to .98 with an effect size estimate (*R*^2^) of .96; the mean correlations for arousal and dominance were .91 (*R*^*2*^ = .83; range of Pearson’s correlation coefficients: .85-.94) and .82 (*R*^*2*^ = .89; range: .64-.89), respectively, suggesting that the ratings had a high internal reliability and can be used across the older Italian population. However, it should be noted that valence ratings were very consistent across older adults, while dominance ratings were more variable and less consistent.

To further test the generalizability of the Italian ANEW adaptation for older adults, we compared our ratings with several smaller sets that had been collected previously by other researchers for populations of a comparable age [20,38,39]. All correlations indicated that our evaluations were all highly consistent with previous studies. In particular, valence in our sample was strongly correlated (*r* = .82-.91, *R*^2^ = .68-.84) with that from other affective norms [20,38,39], showing good generalizability across different languages. Arousal presented more variability than valence with other affective norms [20,38,39], as revealed by lower correlations (*r*: .61-.84, *R*^2^ = .37-.71). Note that for dominance, the correlation between our data and those of Grühn and Smith [38] (the only existing affective norms on older participants that included the dominance dimension) was lower, but significant (*N* = 49, *r* = .55, *p* < .001, *R*^2^ = .31), compared to those found for valence and arousal.

In sum, we found high split-half reliabilities within the older population for all the affective dimensions, indicating a large agreement among older adults. Moreover, these results corroborate those of previous studies in the younger population [11,12,21,22,26], suggesting a strong cross-age reliability (see below -Age-related differences section- for a direct test for the younger-older comparison). Thus, we assume that our norms are reliable and can be confidently used for word selection in emotion research.

More importantly, in agreement with the affective norms on both older [20,38,40] and younger [11,14,26] adults, these results show that valence is more reliable compared to other affective variables, suggesting that valence is a more reliable measure within the Italian language and more stable among different languages compared to the other affective dimensions. A possible account for this result is that valence is an affective dimension existing in all cultures [68] since it is grounded on atavistic motivational brain circuits [69]. For this reason, it is plausible to suppose that the valence concept can be experienced across all ages, and of consequence, its definition is also clearer and univocal compared with those of arousal and dominance.

### Relation between affective variables

#### Valence vs. arousal relation

**Fig 2:**
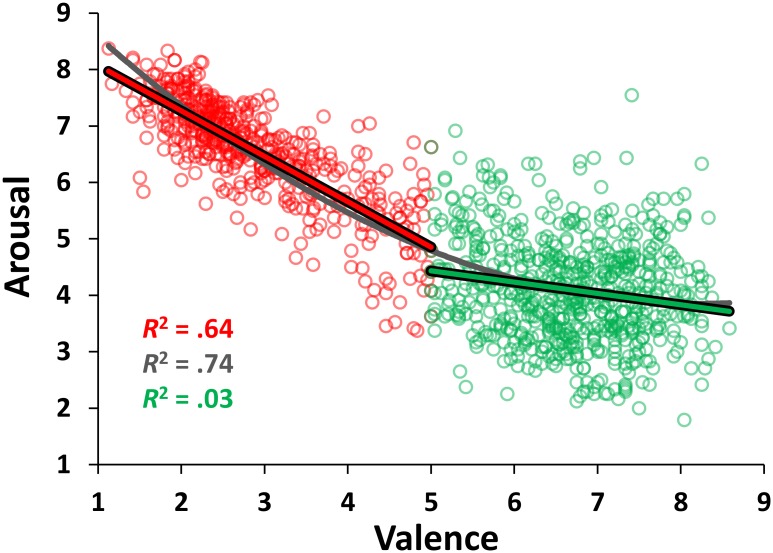
Distribution of the stimuli in the affective space of valence and arousal. The scatterplot shows the mean values for all words of the Italian adaptation of the ANEW in the valence and arousal affective dimensions, along with the quadratic regression line (in dark gray) and the linear regression lines for arousal as predicted by valence above (in green) and below (in red) the median of the rating scale.

After assessing the reliability of our Italian affective norms, we investigated whether the relationship between affective valence and arousal was characterized by the classical U-shaped (i.e., quadratic) function, where the items with lower and higher ratings of valence are perceived to be the most arousing [12]. We performed a regression analysis with mean valence and its square as independent variables and mean arousal as a dependent variable. The resulting quadratic function (y = .091x^2^–1.491x + 9.980) was highly significant, explaining 74.06% of the variance (*r* = .86; *F*_(2, 1118)_ = 1596.3, *p* < .001) (see Fig 2). Importantly, the quadratic function outperformed the simpler linear model (y = -.620x + 8.282), which accounted for 70.64% of the variance (*r* = .84; *F*_(1, 1119)_ = 269.44, *p* < .001) and the *R*^*2*^ change, due to the inclusion of the quadratic term, was highly significant (*F*_(1,1118)_ = 147.40, *p* < .0001), indicating that the quadratic function explains the relationship between valence and arousal better than the linear one.

However, contrary to the previous affective norms on younger participants for words [11,12,14], pictures [70], and sounds [71,72], the relation between valence and arousal did not have a clear U-shape. In other words, despite the fact that the analysis revealed that the quadratic function explained the relationship between valence and arousal better than the linear function, the distribution of affective ratings in the valence by arousal space did not follow the classical boomerang- or U-shape found in previous affective norms in younger participants. Rather, this result is in line with Backs and colleagues [53], who found that the relationship between valence and arousal was less quadratic for older adults.

Moreover, the lack of a clear U-shape is partially in accord with the findings of affective norms in older adults for words [20,38] and pictures [40] (although these latter found a linear relationship between valence and arousal). As pointed out by Gruhn and Scheibe [40], in the domain of a dedifferentiation of intellectual abilities in aging, this result could be due to a decrease of differentiation of affective information processing in older adults—a diminution in “the degree to which the individual is able to differentiate and organize different, and often opposite emotions” [73]. Consistently with a dedifferentiation of intellectual abilities, the aging process might also lead to a degradation and simplification of affective structures [73], with an increase in covariation between valence and arousal. In other words, the different affective dimensions might become less differentiated just as intellectual abilities in aging.

Importantly, a similar trend between our study and previous affective norms in older participants [40,53] was evident for arousal, since older participants rated negative stimuli as more arousing and positive stimuli as less arousing, leading to a less-curved distribution of ratings in the valence by arousal space. In particular, a two-tailed independent *t* test suggested that the negative words (i.e., those with a mean valence score below 5) tended to elicit higher arousal compared to positive ones (i.e., those with a mean valence above 5) (*M*s = 6.54 and 4.09, *SD*s = .93 and .91, respectively; *t*_(1119)_ = 44.17, *p* < .001, Cohen’s *d* = 2.66). Moreover, a categorization of the items as a function of their mean valence ratings in negative (i.e., those with a mean valence score below 3.5; *n* = 362), neutral (i.e., those with a mean valence score between 4.5 and 6.5; *n* = 380), and positive words (i.e., those with a mean valence score above 6.5; *n* = 379) revealed that negative words were rated as much more arousing than neutral ones (*M*s = 6.90 and 4.63, *SD*s = .62 and 1.05, respectively; *t*_(740)_ = 35.74, *p* < .001, *d* = 2.63), which, in turn, were rated as more arousing than positive words (*M* = 3,97, *SD* = .89; *t*_(757)_ = 9.29, *p* < .001, *d* = .68). Notice that this pattern of results remained the same when using different cutoff values to categorize negative, neutral, and positive words. This result is in sharp contrast with what is usually found in younger participants, who rate both negative and positive words with higher arousal values compared to neutral words [11,14,23]. These age-related differences in the distribution of affective ratings could also explain the bimodal distribution found for arousal ratings in older participants (see Descriptive Statistics section), but we will examine this point in more detail below (see Age-related differences section).

More importantly, the relationship between the valence and arousal dimensions seems to be asymmetrical. Indeed, it seems that while arousal strongly increases in relation to the increase of negative valence (y = –.804x + 8.873, *R*^2^ = .64; *F*_(1, 476)_ = 844.26, *p* < .001), it tends to decrease as positive valence increases (y = -.194x + 5.393, *R*^2^ = .03; *F*_(1, 641)_ = 19.99, *p* < .0001) (see Fig 2). An evaluation of the homogeneity-of-slopes assumption indicated that the relationship between the valence and arousal dimensions differs significantly (*F*_(1, 1117)_ = 347.42, *p* = .0001) as a function of item polarity (i.e., the absolute difference between the valence rating and the median of the rating scale), thus confirming the asymmetry described above. However, given the large size of our word set and the small effect size for the relation between positive valence and arousal, this result should be taken with caution. Indeed, by choosing the median of the valence ratings distribution as the cutoff point for the classification in negative and positive words, the relationship between negative words and arousal remains significant (y = –.798x + 8.857, *R*^2^ = .71; *F*_(1, 563)_ = 1407.24, *p* < .0001), whereas that between positive words and arousal is no longer significant (y = –.078x + 4.554, *R*^2^ = .01; *F*_(1, 554)_ = 1.92, *p* = .1665).

These asymmetries are in line with the above mentioned model [67], assuming that valence and arousal are orthogonal, bipolar and independent dimensions. The same pattern of results was observed in ratings for German [17,74], English [14], Finnish [39] and Italian [11] norms with an increase in negative valence accompanied by an increase in arousal. In contrast, the relation between positive valence and arousal seems to be strongly attenuated, since both exciting and calm stimuli can be perceived as highly pleasant. Similar asymmetries have been observed in the assessment of emotional images [40,75]. In addition, Robinson and colleagues [76] posited an affective model with combined effects of valence and arousal. According to this model, positive low-arousal and negative high-arousal stimuli are processed in a special way since they elicit congruent orientation towards a stimulus (approach and withdrawal, respectively) compared with positive high-arousal and negative low-arousal stimuli, eliciting conflicting approach-withdrawal responses [76].

#### Valence vs. dominance relation

Next, we tested the relationship between valence and dominance by performing a polynomial regression with mean valence and its square as independent variables and the mean dominance as a dependent variable. The resulting quadratic function (y = -.056x^2^ + .926x + 2.184) was highly significant, accounting for 71.96% of the variance (*r* = .84; *F*_(2, 1118)_ = 1434.72, *p* < .0001). Moreover, the quadratic model outperformed the simpler linear one (y = .387x + 3.236), which explained 68.67% of the variance (*r* = .82; *F*_(1, 1119)_ = 2452.85, *p* < .0001) and the *R*^*2*^ change due to the inclusion of the quadratic term was significant (*F*_(1,1118)_ = 131.18, *p* < .0001).

**Fig 3:**
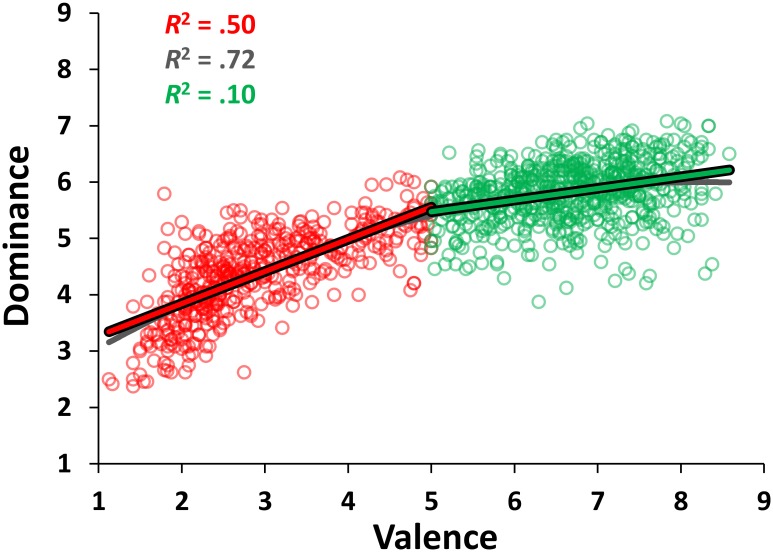
Distribution of the stimuli in the affective space of valence and dominance. The scatterplot shows the mean values for all words of the Italian adaptation of the ANEW in the valence and dominance affective dimensions, along with the quadratic regression line (in dark gray) and the linear regression lines for dominance as predicted by valence above (in green) and below (in red) the median of the rating scale.

However, in this case as well, this relation seems to be asymmetrical, since positive words have higher mean dominance ratings compared to negative words, as confirmed by a two-tailed independent *t* test (*M*s = 5.82 and 4.35, *SD*s = .53 and .74, respectively for positive and negative words; *t*_(1119)_ = 38.47, *p* < .0001; *d* = 2.27). Moreover, the increase in affective dominance was stronger for negative words (y = .570x + 2.704, *R*^2^ = .50; *F*_(1, 418)_ = 475.41, *p* < .0001) than it was for positive words (y = .204x + 4.459, *R*^2^ = .10; *F*_(1, 641)_ = 69.18, *p* < .0001), and word polarity had a significant effect on the slope of the relationship between valence and dominance (*F*_(1, 1117)_ = 469.37, *p* < .0001) (see Fig 3). In this case, despite the very low variance explained by the regression model between positive valence and dominance, the results did not change when choosing the median of the valence ratings as the cutoff point in the classification of negative and positive items. Indeed, the relationship between negative words and dominance continued to be significant (y = .516x + 2.841, *R*^2^ = .60; *F*_(1, 563)_ = 842.13, *p* < .0001), as well as that between positive words and dominance (y = .176x + 4.654, *R*^2^ = .05; *F*_(1, 554)_ = 29.93, *p* < .0001).

Unlike previous studies on younger [11] and older [38] participants, the relation between valence and dominance seems to be explained better by a quadratic relation compared to a linear relation. This result may be due to the fact that the relation between negative words and dominance is steeper than that between positive words and dominance. In line with Fontaine and colleagues [77], we surmise that this result is caused by the fact that negative words can assume opposite values on the dominance scale. Consequently, this factor should be considered useful in discriminating different emotions in the affective space. For example, the words *ira/rage* and *massacro*/*massacre* are both negative words with high arousal, but they take opposite values on the dominance scale. Indeed, in the first case the older participants experienced an “in control” feeling, in the second a “submission” feeling. We believe that these different feelings, which represent two opposite poles of dominance dimension, determine a fight or flight response, respectively in the first and second case. This assumption stems from the original model by Osgood and colleagues [37] corroborated recently [77], which identified dominance as the third independent factor characterizing the emotional space. Within this framework, it should be noted that this asymmetry might be mediated by the different effect of arousal dimension for the positive and negative words.

#### Arousal vs. dominance relation

**Fig 4:**
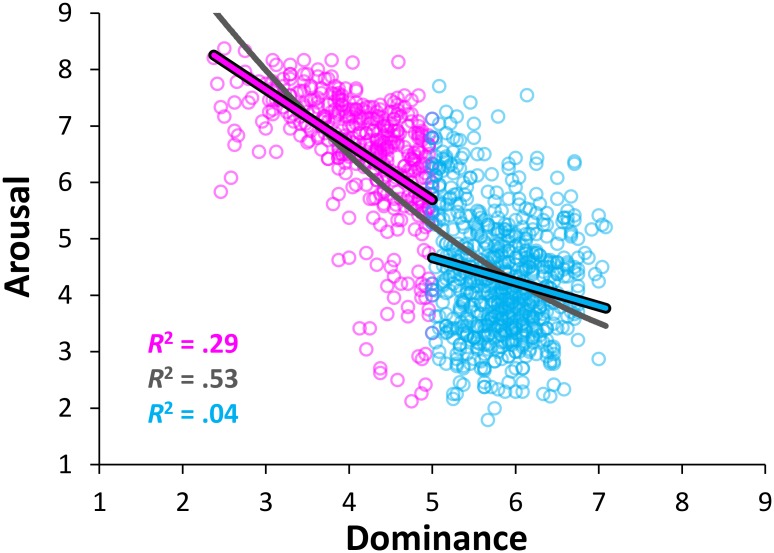
Distribution of the stimuli in the affective space of dominance and arousal. The scatterplot shows the mean values for all words of the Italian adaptation of the ANEW in the dominance and arousal affective dimensions, along with the quadratic regression line (in dark gray) and the linear regression lines for arousal as predicted by dominance above (in cyan) and below (in magenta) the median of the rating scale.

Finally, we investigated the quadratic relation between arousal and dominance by performing a polynomial regression with mean dominance and its square as independent variables and mean arousal as a dependent variable. As for the relations described above, the resulting quadratic function (y = .128x^2^–2.402x + 14.041) was highly significant, accounting for 52.71% of the variance (*r* = .73; *F*_(2, 1118)_ = 623.07, *p* < .0001) (see Fig 4), and outperformed the simpler linear model (y = -1.139x + 11.059), which explained 51.88% of the variance (*r* = .72; *F*_(1, 1119)_ = 1206.523, *p* < .0001). Once again, the *R*^*2*^ change due to the inclusion of the quadratic term was significant (*F*_(1,1118)_ = 19.622, *p* < .0001), albeit the improvement of the explanatory power was negligible (*R*^*2*^ change < .83).

As for the other two relationships, the relation between dominance and arousal was also asymmetrical, since “prevailing” words (i.e., those with a dominance mean below 5) had higher mean arousal ratings than “weak” items (i.e., those with a dominance mean above 5), as confirmed by a two-tailed independent *t* test (*M*s = 6.49 and 4.29, *SD*s = 1.13 and 1.05, for “prevailing” and “weak” words, respectively; *t*_(1119)_ = 33.27, *p* < .0001; *d* = 2.023). Moreover, the increase in emotional arousal was stronger for “prevailing” (y = –.978x + 10.579, *R*^2^ = .29; *F*_(1, 432)_ = 174.65, *p* < .0001) than for “weak” words (y = -.354x + 6.354, *R*^2^ = .02; *F*_(1, 685)_ = 16.91, *p* < .0001), and item polarity had a significant effect on the slope of the relationship between dominance and arousal (*F*_(1, 1117)_ = 135.72, *p* < .0001). As for the relationship between valence and arousal, the results changed when choosing the median of the dominance ratings as the cutoff point in the classification of “prevailing” and “weak” items. Indeed, while the relationship between “prevailing” words and arousal remained significant (y = –1.225x + 11.527, *R*^2^ = .39; *F*_(1, 558)_ = 359.41, *p* < .0001), that between “weak” words and arousal was not significant (y = .108x + 3.512, *R*^2^ = .001; *F*_(1, 559)_ = 1.088, *p* = .2973). Notice that “prevailing” and “weak” stimuli have dominance values below or above 5, respectively, since the affective ratings indicated the participants’ reactions evoked by the stimulus, that is, their own feeling of dominance/control in response to them, rather than the dominant meaning of stimuli.

This result simply reflects the above mentioned fact that emotional space is characterized by three dimensions (or four dimensions, see [77]). The dominance dimension leads to feelings of power or weakness and to the corresponding action tendencies, such as wanting to take initiative versus being apathetic. Arousal, instead, is characterized by activation/readiness for action. In line with this, we suppose that the asymmetry observed might be due to the fact that the “prevailing” stimuli require a greater arousal to prepare an individual to “fight” or “flight”, while “weak” stimuli require a lower activation since the individual is in a “in control” state over the situation. However, these results should be taken with caution because they may be mediated by the relation between the valence and arousal dimensions, since these latter are strongly related, as already mentioned.

### Age-related differences

For the second aim of our study, we tested age-related differences in the word ratings obtained from our current normative study on older adults and those obtained from our previous normative study on younger adults [11].

First, we computed the correlation between the two samples for each affective dimension. Younger and older adults’ ratings were moderately correlated for arousal (*r* = .58) and dominance (*r* = .74), and highly correlated for valence (*r* = .93) dimensions (all *p*s < .0001), suggesting that arousal and dominance ratings are more unstable not only across languages, as shown above (see Reliability of measure section; see also [14,21,26]), but also between younger and older adults [20,53]. Despite this, younger and older adults strongly agree on whether a stimulus was positive, neutral or negative.

Second, we compared the mean affective ratings given by older and younger participants by means of two-tailed paired *t* tests. We found that the average valence ratings were significantly higher for younger than for older adults (*M*s = 5.21 and 5.07, *SD*s = 2.06 and 2.07, respectively; *t*_(1120)_ = 6.406, *p* < .0001), although the effect size was small (*d* = .191). The same result emerged for arousal ratings, but with a greater effect size (*d* = .405), with younger adults reporting to feel significantly more excited by affective words compared to older adults (*M*s = 5.64 and 5.14, *SD*s = .91 and 1.52, respectively; *t*_(1120)_ = 13.543, *p* < .0001). On the contrary, no significant difference emerged between younger and older adults for the dominance dimension (*M*s = 5.23 and 5.20, *SD*s = .98 and .98, respectively; *t*_(1120)_ = 1.557, *p* = .120, *d* = .047). These results were confirmed by further tests investigating possible age-related differences in the distribution of the affective ratings. Indeed, we carried out Kolmogorov-Smirnov tests comparing younger and older distributions of ratings for each affective dimension; results showed that the distribution of dominance ratings was consistent across younger and older participants (*D* = .04, *p* = .296), whereas those of valence and, especially arousal, were significantly different (respectively, *D* = .06 and .32, *p* < .035 and .001). Notice that for the valence dimension, these results seem to be at odd with the lack of significant age-related differences in the percentage of words rated with valence ratings above 5 (see Descriptive Statistics section). However, it should be noted that effect sizes were small and, thus, the significant differences we found between the distributions of valence ratings of younger and older adults could be simply due to the large sample size, and thus should be taken with caution.

Third, we investigated differences between the affective ratings given by older and younger participants for each word. To this end, we performed a series of two-tailed independent *t* tests. The analysis revealed that the older versus younger comparison was significant (*p* < .05) for 287, 487, and 155 words (corresponding to roughly 26%, 43%, and 14% of the total number of words in the database) for valence, arousal and dominance, respectively. A further investigation of these significant comparisons confirmed the findings of the paired *t* tests contrasting mean affective ratings of younger and older participants, as well as those of the Kolmogorov-Smirnov tests contrasting distributions of affective ratings in younger and older participants. In fact, the number of words showing significantly higher valence values for younger than for older participants, as compared to that showing the opposite pattern (186 vs. 101), was significantly higher than chance (binomial test, *p* < .0001). Similarly, a significantly higher number of words showed significantly higher values for younger than older participants, as compared to the opposite pattern, for arousal as well (366 vs. 121; *p* < .0001), again confirming the findings of the analyses reported above. On the contrary, for the dominance dimension, the number of words showing significantly higher values for younger than older participants, as compared to the opposite pattern, was not significantly higher than chance (86 vs. 79; *p* = .074). This again confirmed the findings of the analyses reported above for the dominance dimension, which did not show any significant difference between younger and older participants. Despite the high correlations between younger and older participants, thus, age-related mean differences were evident for the valence and arousal dimensions. Contrary to findings by Grühn and Smith [38], older adults rated words as less positive and more arousing compared to younger adults. It is possible that this discrepancy is because our affective database includes a large number of words with high arousal (74% of total number of words in affective norms on young adults) that, in turn, evoke a feeling of unpleasantness in older adults, linked to their concern about avoiding over-arousal [78]. This might determine a tendency to attribute lower valence scores for negative words in older participants compared to those of young participants. We return to this issue below.

**Fig 5:**
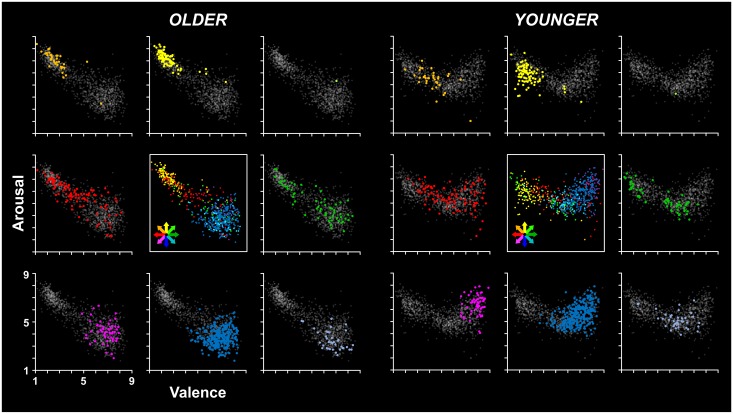
Age-related differences in the distribution of stimuli in the valence by arousal affective space. Each scatterplot shows the mean values for all words of the Italian affective norms in the valence (x-axis) and arousal (y-axis) affective dimensions in both older and younger participants (left and right panel, respectively). In each panel, the colored points shown in the eight external scatterplots represent the words for which a significant difference was found between older and younger adults’ ratings in the valence and/or the arousal dimension, whereas the grey points represent the remaining words. Specifically, the scatterplots along the horizontal and vertical midlines of each panel show, respectively, the words for which the older adults’ ratings differed from the younger ones in either the valence (green and red: higher and lower valence ratings, respectively) or the arousal dimension (yellow and blue: higher and lower arousal, respectively), whereas the scatterplots located in the four corners show the words for which the older and younger adults’ ratings differed in both dimensions (see the text for details). To better illustrate the general pattern of differences between older and younger adults’ ratings in the affective space, the central scatterplot shows all the words showing a significant age-dependent difference (the color code was the same as in the other scatterplots).

Fourth, we investigated whether the above age differences pertain to specific patterns of affective values. To this end, we performed a chi-square test for each pair of affective dimensions (i.e., valence vs. arousal, valence vs. dominance, and dominance vs. arousal) and for both younger and older affective ratings to compare the frequency distribution of the items that showed significantly different ratings between age groups (at the single-word *t* tests for each affective dimension, see previous paragraph) for each of the nine cases deriving from the combination of the older vs. younger contrasts for the two dimensions composing each pair. For example, regarding the valence by arousal distribution of affective ratings, ratings for an item in older adults compared to younger adults, can be either 1) consistent (i.e., non-significantly different at the single-word *t* test) on both valence and arousal dimensions (V = A =), or significantly different in either dimension, specifically (see Fig 5 from the upper right corner, clockwise): 2) higher on both valence and arousal dimensions (V+A+); 3) higher on valence and consistent on arousal dimension (V+A =); 4) higher on valence but lower on arousal dimension (V+A-); 5) consistent on valence and lower on arousal dimension (V = A-); 6) lower on both valence and arousal dimensions (V-A-); 7) lower on valence but consistent on arousal dimension (V-A =); 8) lower on valence but higher on arousal dimension (V-A+); or 9) consistent on valence but higher on arousal dimension (V = A+) (see Fig 5). Moreover, we inspected the pattern of affective values for the same items included in the nine cases.

Regarding the valence by arousal affective ratings, the chi-square test revealed that the distribution of significant across age differences was significantly different from the expected one (*χ*^2^_(4)_ = 23.16; *p* < .0001). In particular, there was a high number of items showing the V-A+ pattern of differences (see orange points in Fig 5). The inspection of these items revealed that they were characterized by a peculiar pattern of valence and arousal ratings by older and younger participants in a consistently different way. Albeit these items were rated by younger participants with middle-to-low valence values and middle-to-high arousal values (see orange points in the right panel of Fig 5), they received more extreme ratings from older participants (i.e., more negative and arousing values), and were located in the upper left corner in the valence by arousal space derived from the older norms (see orange points in the left panel of Fig 5). In other words, while younger participants rated these items as moderately negative and arousing, older participants rated them as very negative and arousing. Similarly, there was another group of items for which younger and older participants provided different arousal ratings, that is, the V = A+ pattern of difference (see yellow points in Fig 5). These items were characterized by low valence ratings and were rated by younger participants with middle-to-high arousal values (and, thus, were clustered in the left part of the corresponding valence by arousal space), but were rated with higher (i.e., more extreme) arousal ratings by older participants, with a consequent upward shift in the corresponding valence by arousal space (see yellow points in the right and left panels of Fig 5).

Moreover, there were two other groups of words showing specific patterns of valence and arousal ratings by older and younger participants in a consistently different way, namely the V-A- and, especially, the V = A- words (see magenta and blue points, respectively, in Fig 5). These items were rated by younger participants as generally positive (i.e., with valence values higher than five) and with middle and middle-to-high arousal values, so that they were clustered in the right part in the valence by arousal space derived from the younger norms (see magenta and blue points in the right panel of Fig 5); conversely, these words were consistently rated by older adults as less arousing, with a global downward shift in the location of these items in the valence by arousal space derived from the older norms (see magenta and blue points in the left panel of Fig 5). This provides direct support for the impression that older participants rated negative stimuli as more arousing and positive stimuli as less arousing, thus leading to a less-curved distribution of ratings in the valence by arousal space. Importantly, the age-related differences in the distribution of affective ratings in the valence by arousal space described above could also explain the bimodal distribution found for arousal ratings in older participants. Indeed, the age-related increase of arousal ratings for mid-negative words, which had middle-to-high arousal ratings in younger, along with the age-related reduction of arousal ratings for positive words, which had middle and middle-to-high arousal ratings in younger, would have caused the distribution of arousal ratings to become bimodal in older participants.

**Fig 6:**
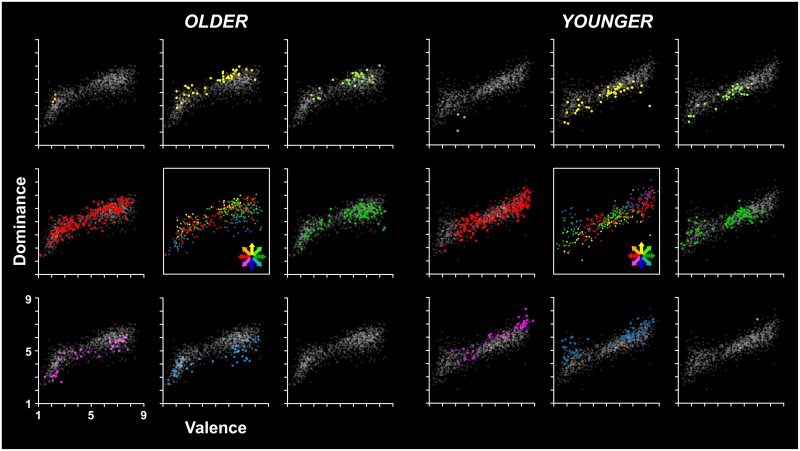
Age-related differences in the distribution of stimuli in the valence by dominance affective space. Each scatterplot shows the mean values for all words of the Italian affective norms in the valence (x-axis) and dominance (y-axis) affective dimensions in both older and younger participants (left and right panel, respectively), In each panel, the colored points shown in the eight external scatterplots represent the words for which a significant difference was found between older and younger adults’ ratings in the valence and/or the dominance dimension, whereas the grey points represent the remaining words. Specifically, the scatterplots along the horizontal and vertical midlines of each panel show, respectively, the words for which the older adults’ ratings differed from the younger ones in either the valence (green and red: higher and lower valence ratings, respectively) or the dominance dimension (yellow and blue: higher and lower dominance, respectively), whereas the scatterplots located in the four corners show the words for which the older and younger adults’ ratings differed in both dimensions (see the text for details). To better illustrate the general pattern of differences between older and younger adults’ ratings in the affective space, the central scatterplot shows all the words showing a significant age-dependent difference (the color code was the same as in the other scatterplots).

With regards to the valence by dominance affective ratings, the chi-square test revealed that the distribution of significant across-age differences was significantly different from the expected one (*χ*^2^_(4)_ = 126.17; *p* < .0001). In particular, there was a high number of items showing V+D+ and V-D- patterns of differences (see light green and magenta points, respectively, in Fig 6). However, the inspection of affective ratings for these items revealed that the valence and dominance ratings provided by both younger and older participants for these items were somehow variable, with no apparent pattern (see Fig 6).

**Fig 7:**
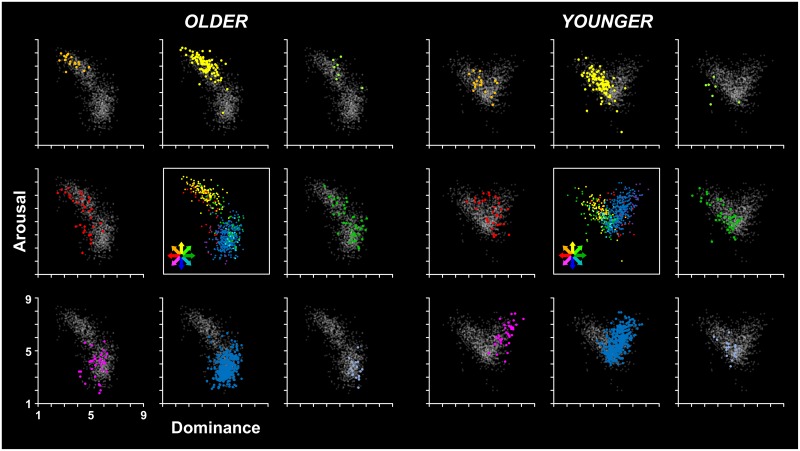
Age-related differences in the distribution of stimuli in the dominance by arousal affective space. Each scatterplot shows the mean values for all words of the Italian affective norms in the dominance (x-axis) and arousal (y-axis) affective dimensions in both older and younger participants (left and right panel, respectively), In each panel, the colored points shown in the eight external scatterplots represent the words for which a significant difference was found between older and younger adults’ ratings in the dominance and/or the arousal dimension, whereas the grey points represent the remaining words. Specifically, the scatterplots along the horizontal and vertical midlines of each panel show, respectively, the words for which the older adults’ ratings differed from the younger ones in either the dominance (green and red: higher and lower dominance ratings, respectively) or the arousal dimension (yellow and blue: higher and lower arousal, respectively), whereas the scatterplots located in the four corners show the words for which the older and younger adults’ ratings differed in both dimensions (see the text for details). To better illustrate the general pattern of differences between older and younger adults’ ratings in the affective space, the central scatterplot shows all the words showing a significant age-dependent difference (the color code was the same as in the other scatterplots).

With regards to the dominance by arousal affective ratings, the chi-square test revealed that the distribution of significant across-age differences was significantly different from the expected one (*χ*^2^_(4)_ = 19.34; *p* = .001). In particular, there was a high number of items showing the D-A+ pattern of differences (see orange points in Fig 7). The inspection of these items revealed that they were characterized by a peculiar pattern of dominance and arousal ratings that resembles that observed for the valence by arousal analysis. Albeit these items were rated by younger participants with middle dominance and arousal values (see orange point in the right panel of Fig 7), they received more extreme ratings from older participants on both dimensions (i.e., lower dominance and higher arousal values). That is, they were located in the upper left corner in the dominance by arousal space derived from the older norms (see orange point in the left panel of Fig 7). In other words, while younger participants rated these items as moderately prevailing and arousing, the older participants rated them as very prevailing and arousing.

There were three other groups of items that were characterized by peculiar patterns of dominance and arousal ratings that resemble that observed for the valence by arousal analysis, namely the items showing the D = A+, D-A-, and D = A- patterns of age-related differences (see, respectively, yellow, magenta, and blue points in Fig 7).

The results thus showed that, despite the high consensus with respect to the ratings of emotional words, as evidenced by the high correlations between younger and older adults’ affective ratings, a major number of words revealed age-related differences. Partially in line with the study by Grühn and Scheibe [40], older adults rated the middle-low valence and arousing words as more negative and arousing compared with younger adults did. This suggests that the older adults have a tendency to respond more extremely only to negative and arousing words. Unlike previous study [40], we did not find a clear pattern of extreme ratings for positive words. This could be due to the fact that word stimuli elicit the emotions with less intensity compared with the pictures adopted in the Grühn and Scheibe’s study [40]. More importantly, Keil and Freund [49] observed that in old age, the appetitive activation –i.e., the relationship between arousal and pleasure ratings [79]- generally decreased. In contrast, the aversive activation –i.e., the increase of emotional arousal with unpleasantness –increased with age [49]. For example, Bradley and Lang [80] have reported that words rated with high arousal values took more negative scores for older women. Moreover, a relation between displeasure and arousal increased as a linear function of age [49]. According to the authors, in late adulthood, high arousal is experienced as negative since the regulation of the physiological system might become more difficult and last longer before reaching an optimal activation state. For this reason, these deviations from the optimal state might be experienced as unpleasant. An alternative explanation is that on the one hand, the negative stimuli per se receive higher arousal and lower dominant ratings and elicit a stronger response compared with positive stimuli [81]; on the other hand, there is an age-related increase in the self-reported affective intensity [53,54]. Together, these two factors could have determined this difference between younger and older adults.

Moreover, as already mentioned above, the relation between the valence and arousal dimensions is mediated by the dominance dimension. Going beyond previous studies [40,52,82], we also found that words rated with low dominance and arousal by younger adults were rated with very extreme values by older adults, proposing that older participants have more intense feeling of submission. This tendency was also evident in the correlation between words with low dominance and arousal, as described above.

Together, our findings suggest that older adults consider words that younger adults rated with middle scores for valence, dominance and arousal as less dominant but more arousing and negative compared to younger adults. In our view, these results should be framed within a three-dimensional affective space given by three orthogonal axes of valence, arousal and dominance, in line with the original model of emotion [37], since the bi-dimensional space, characterized by valence and arousal dimensions, might fail to account for important sources of variance. One way of interpreting these differences between younger and older adults might be attributed to the fact that the older adults might be deficient in a sense of control due to lower feeling of efficacy related to physical and environmental limitations. For this reason, for example, words related to health problems as *cancro/cancer* could be perceived as more negative, less controllable and arousing since older adults perceive these problems as not controllable. However, this account is highly speculative and merits further investigation.

## Conclusion

The main aim of this study was to provide an empirical validation of an affective word database for older adults. Affective ratings were collected from a large sample of Italian older adults in order to provide information about the affective connotation that older adults’ attribute to verbal stimuli. The rationale was the following. First, we wanted to systematically validate our previously developed database with older adults. The secondary aim was to investigate how the processing of affective information of words changes with age.

In line with the dimensional theory of emotion, we provided affective ratings for valence, arousal and dominance for Italian words in older adults. We found that although ratings were reliable within old sample and, especially for the valence dimension, also across-languages and between younger and older adults, some discrepancies with previous studies have been emerged. Moreover, there were age-related differences for a great number of words. Older adults tended to give more extreme valence and arousal ratings for arousing words with middle-low scores on the valence scale than younger adults. Moreover, they also rated positive stimuli as less arousing than younger participants. This pattern of age-related differences in affective ratings determined a more pronounced linear relationship between negative words and arousal than that between positive words and arousal and led to a less-curved distribution of ratings in the valence by arousal space. A similar pattern was evident for the relationship between dominance and arousal. For these reasons, it is recommended to consider age- and language-related differences when using the Italian affective norms for words in older participants across languages and age groups.

In light of previous research, it would also be important to collect semantic [83–85] and lexical [86,87] indexes to investigate their relation with the affective properties of words. Therefore, future research could expand the present database to include these variables. Nevertheless, this study provides useful measures that allows researchers to select affective words matched on affective dimensions of valence, arousal and dominance across younger and older participants for future research on aging. To the best of our knowledge, our investigation constitutes the first attempt to develop a comprehensive word dataset for older adults in Italian. Further studies will need to extend these results to pictorial material as well and compare the attribution of an emotional connotation across stimuli in the Italian older adult population aimed to facilitate the sharing of a common methodology and study comparisons in aging and emotion literature.

## Supporting Information

S1 Database Affective ratings for the Italian adaptation of the ANEW stimuli. The supplementary material includes the affective ratings instruction in the Instructions sheet of the S1 Database file (XLSX). It includes also the database with the valence, arousal, and dominance ratings for the whole sample of older participants, as well as the statistics of the comparisons between older and younger participants for the valence, arousal and dominance ratings of each word. The contents of the database are described in more detail in the Description sheet of the S1 Database file.
(XLSX)
